# Comparison of CD146 +/− mesenchymal stem cells in improving premature ovarian failure

**DOI:** 10.1186/s13287-022-02916-x

**Published:** 2022-06-21

**Authors:** Lin Zhang, Yang Sun, Xiao-Xu Zhang, Yu-Bin Liu, Hui-Yan Sun, Chu-Tse Wu, Feng-Jun Xiao, Li-Sheng Wang

**Affiliations:** 1 Beijing Institute of Radiation Medicine, Beijing, 100850 People’s Republic of China; 2 Laboratory of Molecular Diagnosis and Regenerative Medicine, Medical Research Center, The Affiliate Hospital of Qingdao University, Qingdao, 266000 People’s Republic of China; 3 Yanda Medical Research Institute, Hebei Yanda Hospital, Sanhe, 065201 Hebei Province People’s Republic of China

**Keywords:** UC-CD146 +/− MSCs, POF, Immune regulation

## Abstract

**Background:**

Mesenchymal stem cells (MSCs) are a heterogeneous group of subpopulations with differentially expressed surface markers. CD146 + MSCs correlate with high therapeutic and secretory potency. However, their therapeutic efficacy and mechanisms in premature ovarian failure (POF) have not been explored.

**Methods:**

The umbilical cord (UC)-derived CD146 +/− MSCs were sorted using magnetic beads. The proliferation of MSCs was assayed by dye670 staining and flow cytometry. A mouse POF model was established by injection of cyclophosphamide and busulfan, followed by treatment with CD146 +/− MSCs. The therapeutic effect of CD146 +/− MSCs was evaluated based on body weight, hormone levels, follicle count and reproductive ability. Differential gene expression was identified by mRNA sequencing and validated by RT-PCR. The lymphocyte percentage was detected by flow cytometry.

**Results:**

CD146 +/− MSCs had similar morphology and surface marker expression. However, CD146 + MSCs exhibited a significantly stronger proliferation ability. Gene profiles revealed that CD146 + MSCs had a lower levels of immunoregulatory factor expression. CD146 + MSCs exhibited a stronger ability to inhibit T cell proliferation. CD146 +/− MSCs treatment markedly restored FSH and E2 hormone secretion level, reduced follicular atresia, and increased sinus follicle numbers in a mouse POF model. The recovery function of CD146 + MSCs in a reproductive assay was slightly improved than that of CD146 - MSCs. Ovary mRNA sequencing data indicated that UC-MSCs therapy improved ovarian endocrine locally, which was through PPAR and cholesterol metabolism pathways. The percentages of CD3, CD4, and CD8 lymphocytes were significantly reduced in the POF group compared to the control group. CD146 + MSCs treatment significantly reversed the changes in lymphocyte percentages. Meanwhile, CD146 - MSCs could not improve the decrease in CD4/8 ratio induced by chemotherapy.

**Conclusion:**

UC-MSCs therapy improved premature ovarian failure significantly. CD146 +/− MSCs both had similar therapeutic effects in repairing reproductive ability. CD146 + MSCs had advantages in modulating immunology and cell proliferation characteristics.

**Supplementary Information:**

The online version contains supplementary material available at 10.1186/s13287-022-02916-x.

## Introduction

Mesenchymal stem cells (MSCs) have been recognized as major therapeutics and widely applied in regenerative medicine [1]. Preclinical and clinical studies of MSCs exhibit their therapeutic effects in a broad field of disorders and injuries including wound healing [2], immune and ischemia diseases [3, 4], soft tissue defects, hair loss [5] and covid-19 [6]. Various types of MSCs derived from different resources such as umbilical cord-derived MSCs(UC-MSCs) [7], adipocyte-derived MSCs(Ad-MSCs) [8] and bone marrow-derived MSCs(BM-MSCs) [9] have been applied in the treatment of a variety of refractory diseases, including tissue damage and immune diseases. However, the MSC therapy still had limitation in clinical application for treatment of some diseases because of their heterogeneity in cell subpopulation and regenerative ability. The precise MSC therapy is urgent to be developed.

Mesenchymal stem cells express CD105, CD73, CD44, and CD90. They do not express the hematopoietic markers such as CD45, CD34, CD11b, CD19, or HLA-DR [10]. However, the MSCs are a heterogeneous group of subpopulations which express differential surface markers and possess differential biological characteristics [11]. Thus isolation and characterization of MSCs subpopulations from various sources, in order to assess their potential and define their therapeutic functions for diseases, will be the future direction for precise stem cell therapy.

A number of candidate MSCs surface markers, including Stro-1, SSEA-4, CD271, and CD146, are potentially related to their stemness and biological function [11]. CD146, also known as melanoma cell adhesion molecule (MCAM), is a membrane glycoprotein. It is also a member of the immunoglobulin gene superfamily which was founded in human melanoma cells [12]. CD146 has also been validated to have a high expression in several types of cells including vascular endothelial cells, smooth muscle cells, and pericytes from multiple tissues. It plays important roles in vascular endothelial cell activity and angiogenesis [13, 14]. Notably, CD146 emerges as an attractive candidate surface marker to distinguish the particular MSC subgroup [15]. The CD146 + MSCs subgroup exhibits superior biological activity and therapeutic potential in regenerative medicine [16–18]. Bone marrow-derived CD146 + MSCs are significantly pronounced in migration and homing abilities [19–21]. More importantly, CD146 + BM-MSCs show biological characteristics of stronger immune inhibition and cytokine secretion [22]. CD146 expression is significantly associated with the proliferation and angiogenesis activity in AD-MSCs [23, 24]. Transplantation of CD146 + AD-MSCs could prolong the survival period of mice suffering from muscular atrophy, which is due to their ability to regulate the immune system and promote angiogenesis [25]. Dental pulp tissue (DP)-derived CD146 + MSCs exhibit stronger osteogenic differentiation ability than CD146-MSCs [26]. Their differences in proliferation and immune regulatory ability are associated with ERK/p–ERK pathway [27]. Aged MSCs express low level of CD146 and exhibit weak proliferation and differentiation ability. CD146 could act as a useful marker for predicting senescence of MSCs and be applied in quality-control assessments of MSC-based therapy [28].

Premature ovarian failure (POF) is a syndrome of losing ovarian function in younger women usually below the age of 40. Women with POF exhibit disease characteristics of infertility, lower estrogen levels and higher gonadotropin concentrations [29]. The incidence of POF worldwide is 1–3% and has gradually increased in recent years [30, 31]. MSCs have been applied for treatment of POF and achieved satisfactory therapeutic effects from a variety of sources including bone marrow, umbilical cord, placenta, amniotic and adipose tissue [32–34]. Long et al. treated POF patients with UC-MSCs to deliver healthy fetuses successfully, providing the direct evidence that MSCs treatment can be effective in POF [35]. In this study, the CD146 +/− MSCs were isolated by magnetic beads and  their biological characteristics and therapeutic efficacy in a mouse POF model were compared.

## Materials and methods

### Cell culture

Human umbilical cord-derived mesenchymal stem cells (UC-MSCs) at passage 1 were provided by the Allcare Biomedical Development Co. LTD (Qingdao, China). The cells were cultured with 90% minimum essential medium (αMEM, BI) and 10% fetal bovine serum (Gibco, Australia) at 37 °C with 5% CO_2_. After the further passage (P), MSCs were harvested with trypsin–EDTA (Gibco, Australia), counted, and then reseeded in 100 mm cell culture dishes at a density of 20,000 cell/cm^2^.

### Isolation of CD146 + MSCs and CD146 - MSCs

UC-MSCs (three different donors) were separated into CD146 +/− subpopulations using magnetic-activated cell sorting (MACS; MiltenyiBiotec Inc., BergischGladbach, Germany). Cells were incubated with FcR Blocking Reagent and CD146 MicroBeads (CD146 MicroBead Kit; MiltenyiBiotec Inc.) and then underwent magnetic separation. LS Columns were used for CD146 + cells selection, and the CD146 - cells from LS columns were separated again by LD column to increase the purity of CD146 - cells. The separated cells were incubated with APC-conjugated human CD146 antibodies (Biolegend, San Diego, USA) for 15 min at room temperature. The percentage of CD146-positive cells was measured by flow cytometry (Beckman, cytoflexs).

### Phenotypic characterization

4 × 10^5^ cells were divided into two tubes, and antibodies CD45-PerpCP, CD34-PI, CD90-PE (all Biolegend) and CD73-PE (Biolegend), CD105-Alexa Fluor647 (eBioscience, Thermo Fisher, USA) were added to the two tubes respectively and incubated for 30 min at 4 °C. After washing with PBS + 0.5%FBS twice, cell percentages were detected by flow cytometry (Beckman, cytoflexs).

### Cell proliferation

2 μL 10 μM cell proliferation dyeFluor® 670 (eBioscience, Thermo Fisher, USA) was added into 1 mL PBS and mixed with an equal volume of 40,000 cells/mL cell suspension. The staining system was incubated at 37 °C for 10 min in the dark. Four volumes of pre-cooled medium (containing 10%FBS) was added to stop labeling. After incubation for 5 min on ice, cells were plated in 6-well plates at 1 × 10^5^ cells /well. Four time points of 0 h, 24 h, 48 h, and 72 h post-culture were set for flow cytometry detection. The CD146 + MSCs were stained with CD146-FITC antibody (Biolegend, San Diego, USA) and gated in the assay.

### Co-culture of mesenchymal stem cells with T lymphocytes

The pLKO.1-Luc plasmid, carrying luciferase reporter gene and puromycin-resistance gene, was packed into lentivirus. The mononuclear cells of peripheral blood were separated using human lymphocyte separation solution. T cells were prepared with the magnetic bead sorting kit (Invitrogen, USA). After the cells were transfected with luciferase lentivirus for 48 h, 2 μg/mL puromycin was added for 3 days to select the transfected T cells. The CD146 +/− MSCs were seeded to a six-well plate at a concentration of 2 × 10^5^ cells/well. After the MSC adhered for 6 h, 1 μL of 20 mg/mL mitomycin C was added into the wells for 30 min. After washing three times with PBS, 1 × 10^6^ luciferase T cells were added and cultured for 1–3 days. At 24 h, 48 h, and 72 h post-culture, the luciferase T cells were collected to evaluate their proliferation by luciferase activity assay. 100 μL ONE-GloTM (Promega, USA) reagent was added directly into the culture and incubated for ten minutes at room temperature. Then luminescence was measured with a Spectra Max i3X (Molecular Devices).

### Mouse POF model

All animal experiments were approved by the Institutional Animal Care and Use committee of the Beijing Institute of Radiation Medicine. To establish the premature ovarian failure model, 6–8-week-old female C57BL/6 mice in the experimental group were injected subcutaneously with busulfan (Bu, 30 mg/kg, dissolved in DMSO) and intraperitoneally with cyclophosphamide (Cy, 120 mg/kg, dissolved in normal saline), while mice in the control group (*n* = 20) were injected with equal volumes of DMSO and normal saline. In the POF group (*n* = 20), the mice received no cell therapy after chemotherapy. In the CD146 +/− MSCs-treated groups (*n* = 20), one week after chemotherapy, 1 × 10^6^ CD146 +/− MSCs in 100 μL PBS were injected into mice via the tail vein. The injection was repeated the following week. Animals were sacrificed at 2 W or 4 W after the first cell transplantation. After the second injection of CD146 +/− MSCs, adult males of proven fertility were housed with the females at a ratio of 1:2. The number of litters per pregnancy was recorded.

### Bioluminescence imaging

The bead-sorted CD146 +/− MSCs were infected with 200 MOI luciferase adenovirus. After 36 h, the POF mice were injected with the CD146 +/− MSCs via tail vein and bioluminescence imaged at 3 h post-injection (d0), d3 and d6. The NightOWL LB983 (Berthold, Germany) was used to obtain images of bioluminescence. 10 to 15 min prior to imaging, mice were injected intraperitoneally with D-luciferin (Yeasen Biotechnology, Shanghai, China) at a dose of 150 mg/kg body weight in DPBS and then sedated with 2.5% isoflurane in oxygen. Images were obtained with field-of-view 11.2 cm, binning3.0*3.0, and automatic exposure time. Images were analyzed using Indigo imaging software ver. A 01.19.01.

### RNA sequencing

Total RNA of three pairs of CD146 +/− MSCs derived from 3 donors and twelve ovarian tissues that came from 4 groups was extracted according to the instruction manual of the Trizol Reagent (Life technologies, California, USA). RNA integrity and concentration were checked using an Agilent 2100 Bioanalyzer (AgilentTechnologies, Inc., Santa Clara, CA, USA). The cDNA library was constructed following the manufacturer’s instructions of NEBNext Ultra RNA LibraryPrep Kit for Illumina (NEB, E7530) and NEBNext Multiplex Oligos for Illumina (NEB, E7500). The cDNA libraries were loaded on an Illumina HiSeq sequencing platform at Biomaker (Beijing, China). The adapters and reads from raw reads of each sample were trimmed to obtain clean reads. The clean reads were mapped against the human genome (Homo sapiens; GRCh38) or (Musmusculus.GRCm38) using HISAT2 v2.0.4. Gene expression levels were estimated using fragments per kilobase of exon per million fragments mapped (FPKM). The false discovery rate (FDR) control method was used to identify the threshold of the *P*-value in multiple tests in order to compute the significance of the differences. Significantly differential expression was accepted as |log2FC|> 1.5 and *P*-value < 0.01. Functional annotation and enrichment analysis of the significantly differentially expressed genes was performed with a bioinformatic pipeline tool, BMK Cloud online platform.

### Ovary and spleen histological analysis

At 4 weeks post-MSC therapy, ovaries were collected and fixed with 4% formaldehyde. The ovaries were cut into 5-μm slices and stained with H&E for histological evaluation by light microscopy. The follicles were counted and recorded as preantral follicles (primordial, primary), antral follicles (secondary, mature), or atresia follicles. The spleens were treated in the same way as the ovaries for H&E staining. The images were taken under a light microscope and histological changes of the spleens were recorded.

### Immunohistochemical analysis

After being incubated with 5% bovine serum albumin for 30 min to block the nonspecific antibody binding sites, the samples were then incubated with the primary antibodies against Ki67 (1:500 dilution; Abcam), FABP4 (1:5000 dilution; Abcam), and Adiponectin (1:4000 dilution; Abcam) separately over-night at 4 °C. Next, sections were incubated with corresponding HRP-labeled secondary antibodies (Servicebio; 1:200), and the immunoreactivity was visualized with 0.05% diaminobenzidine (DAB, Servicebio).

### Enzyme-linked immunosorbent assay

Two and four weeks after cell transplantation, blood samples were obtained from eyeball veins and centrifuged at 3000 rpm for 15 min. FSH, E2, AMH,IL-6,IL-2 and TNF-α levels in the serum were measured with an ELISA kit (LIANKE BIOTECH, Hangzhou, China) according to the manufacturer’s instructions.

### Lymphocyte percentage in peripheral blood

Retro-orbital blood samples were obtained from the mice, heparin was used for anticoagulation, and samples were stained with PE-Cy7-CD3 antibody (Biolegend), APC-H7-CD8 (2 W) or Percp-CD8 (4 W) antibody (Biolegend), and V450-CD4 (2 W) and Alexa-Fluor-CD4 (4 W) antibodies (eBioscience). After incubation for 30 min at room temperature, the erythrocytes were lysed with red blood cell (RBC) lysis buffer (Biolegend) and then analyzed by flow cytometry.

### Real-time PCR analysis

Total RNA was extracted from the ovaries using RNAiso Plus (Takara, Beijing, China). The cDNAs were generated using EasyScript^R^One-step gDNA Removal and cDNA Synthesis SuperMixKit (Transgene, Beijing, China) according to the manufacturers’ instructions. Real-time PCR was set up in triplicate using the SYBR Green PCR Master Mix (QIAGEN, Germany) and run on Applied Biosystems Q5. The primer sequences were designed based on the NCBI database. Ratios of gene expression were displayed as fold-change relative to the control group after normalizing to β-actin, the internal standard gene. Data were analyzed with the 2 ^−ΔΔCt^ method.

### Data analysis

Data are expressed as mean ± standard deviation (SD). All analyses were performed using GraphPad Prism software. Independent-sample t tests and ordinary one-way analysis of variance (ANOVA) were carried out for two-group and multiple-group comparisons, respectively. Post hoc tests were performed using the Tukey HSD (homogeneity of variance) test or Dunnett T3 (heterogeneity of variance) test. *P* < 0.05 was considered statistically significant.

## Results

### The biological characteristics of UC-CD146 +/− MSCs

**Fig. 1:**
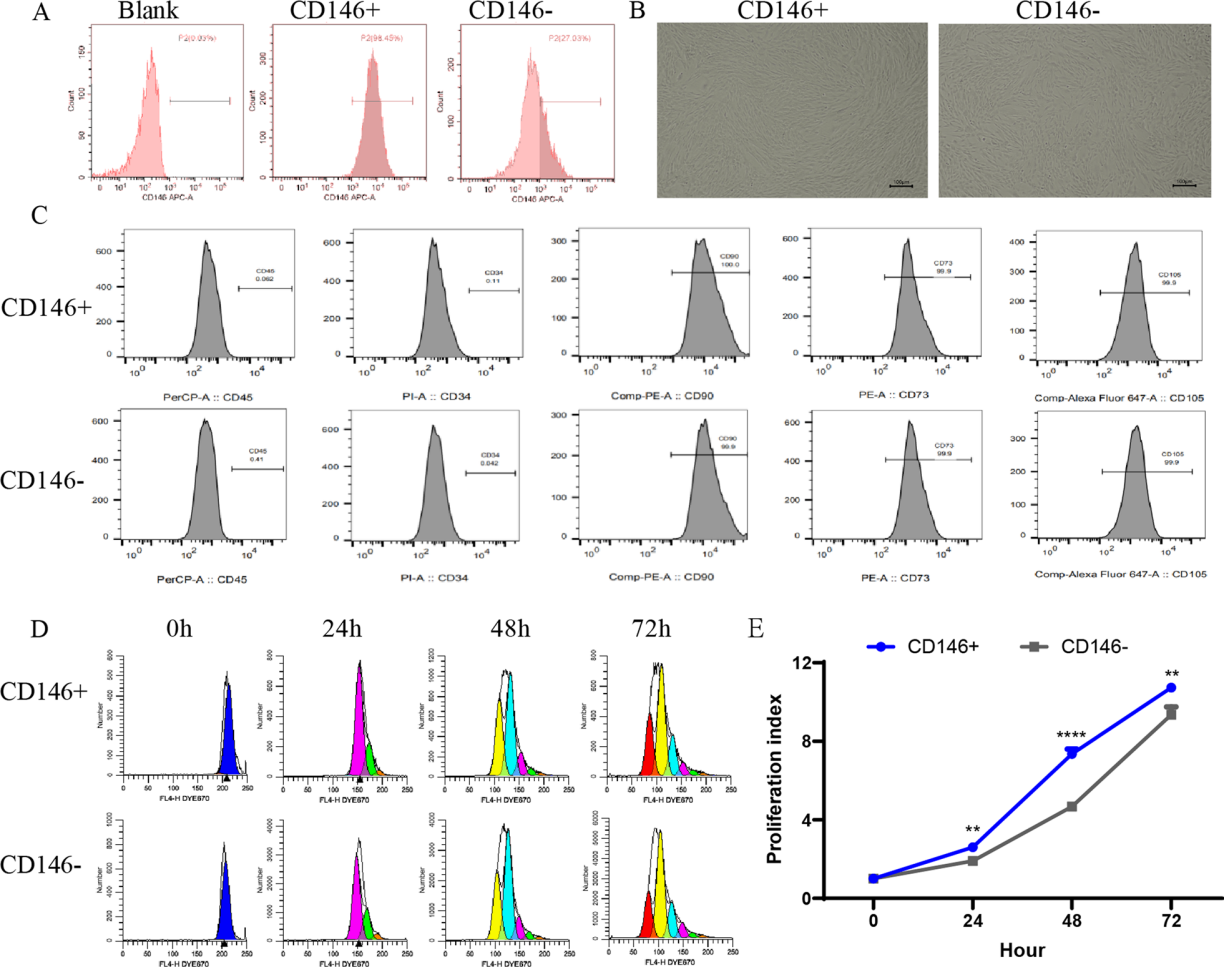
Comparison of biological characteristics of CD146 +/− MSCs subpopulations. **A** Flow Cytometry detection for CD146 expression in UC-MSCs after separation using magnetic beads. **B** The cellular morphology of CD146 +/− subsets after separation using magnetic beads. **C** Flow Cytometry detection of surface markers of CD146 +/− MSCs subsets: CD105, CD73, and CD90 were positive, and CD45 and CD34 were negative. **D** CD146 +/− MSCs subsets were labeled with Dye eFluor™ 670, and cell proliferation was assayed by flow cytometry at 0 h, 24 h, 48 h and 72 h. **E** Cell proliferation index calculated based on flow experimental results

The UC-MSCs were cultured and identified based on morphology and surface markers. UC-CD146 +/− MSCs were separated using the immune magnetic bead system, and their purity was analyzed with flow cytometry. The percentages of CD146 + MSCs before separation are ranged from 70 to 80% (Additional file 1: Figure S1). The percentage of CD146 + MSCs are stably maintained several passages (Additional file 1: Figure S1). The percentage of CD146 + MSCs subpopulation after separation reached 98% (Fig. 1A). The morphology of CD146 +/− MSCs were investigated under a microscope. Both CD146 + and CD146 − subgroups had similar morphological characteristics such as single-layer adherence and a long, spindle-like phenotype (Fig. 1B). They expressed high levels of MSCs surface markers such as CD105, CD73, and CD90. These MSCs did not express the hematopoietic markers such as CD19, CD45or CD34 (Fig. 1C). After eFluor® 670 staining for a proliferation assay (Fig. 1D), the CD146 + MSCs exhibited significantly higher proliferative ability than CD146 - MSCs (Fig. 1E).

### CD146 + MSCs showed lower immunoregulatory factor level and higher T cell inhibition ability

**Fig. 2:**
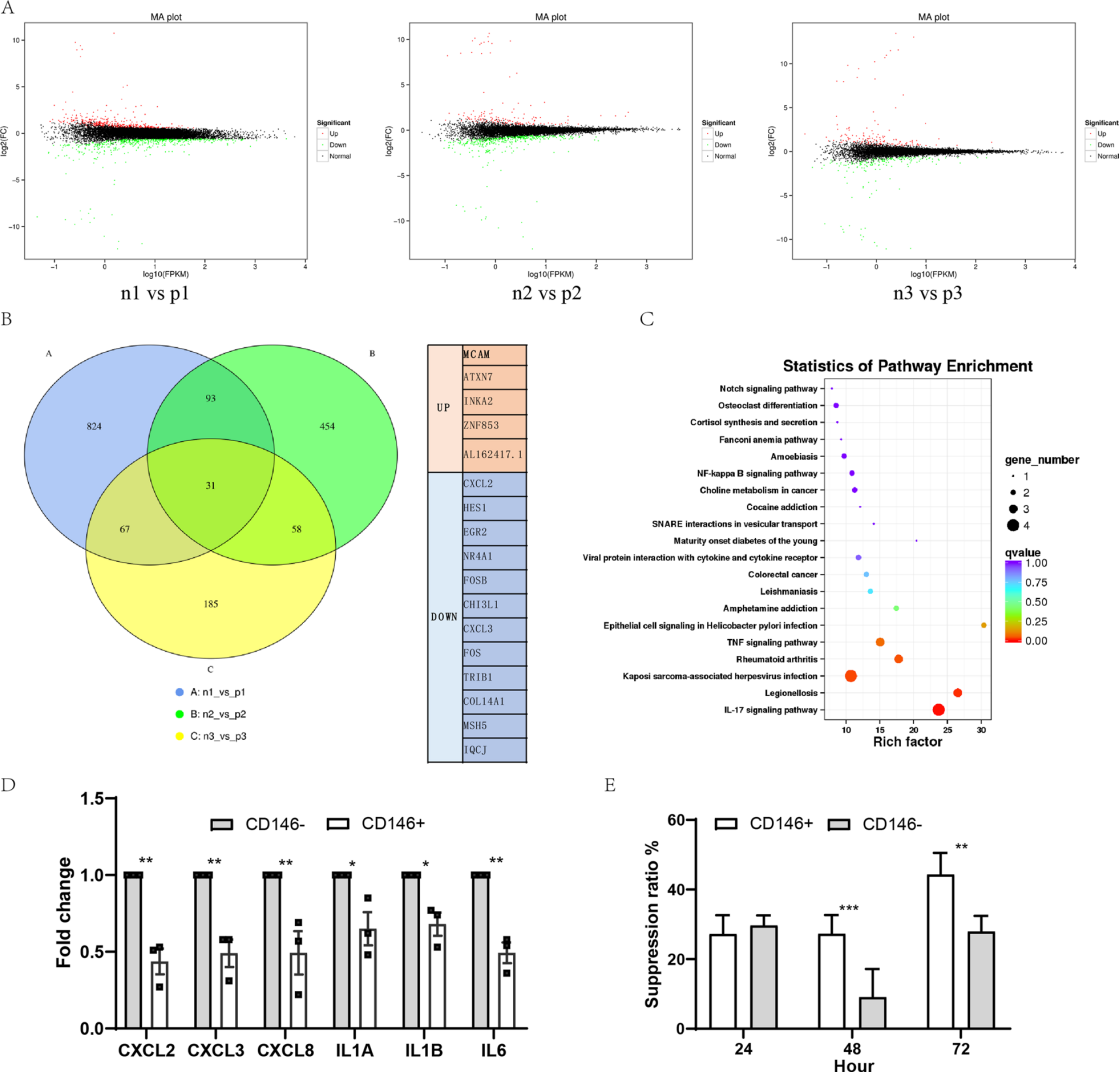
Comparison of differential expressed genes in CD146 +/− MSCs subgroup. **A** Gene expression MA diagram of three pairs of CD146 +/− subpopulations. **B** Venn diagram shows common differential genes of three pairs of CD146 +/− subpopulations and table shows specific common differential genes. **C** KEGG pathway enriched by common differential genes. **D** qPCR validated partially enriched immuno-related differential genes. **E** Co-culture of CD146 +/− subpopulations and luciferase T cells to detect the inhibition of T cell proliferation

To compare the differences in gene expression levels of CD146 +/− subsets, we performed RNA sequencing on three pairs of CD146 +/− MSCs specimens. The MA-plot showed the overall differential gene distribution (Fig. 2A). There were 31 differential genes in common among the three pairs of specimens, including some chemokines and inflammation genes (Fig. 2B). KEGG analysis revealed that major pathways for differential gene enrichment involved in inflammatory disease (Fig. 2C). The qPCR results validated the difference genes of IL-6, IL-1A, and IL-1B as well as CXCL-2, CXCL-3, and CXCL-8. We found that expression levels of these genes in CD146 + MSCs were significantly lower than in CD146 - MSCs (Fig. 2D), suggesting that CD146 +/− subgroups had different immunomodulatory abilities. The inhibitory ability of both subsets on T cell proliferation in vitro was further examined. Compared with the CD146 - MSCs group, the CD146 + MSCs group significantly inhibited T cell proliferation (Fig. 2E). The above results indicated that the CD146 + MSCs had stronger immunomodulatory ability.

### The effect of CD146 +/− MSCs treatment on body weight, mouse progeny, organ coefficient, and distribution assay in a mouse POF model

**Fig. 3:**
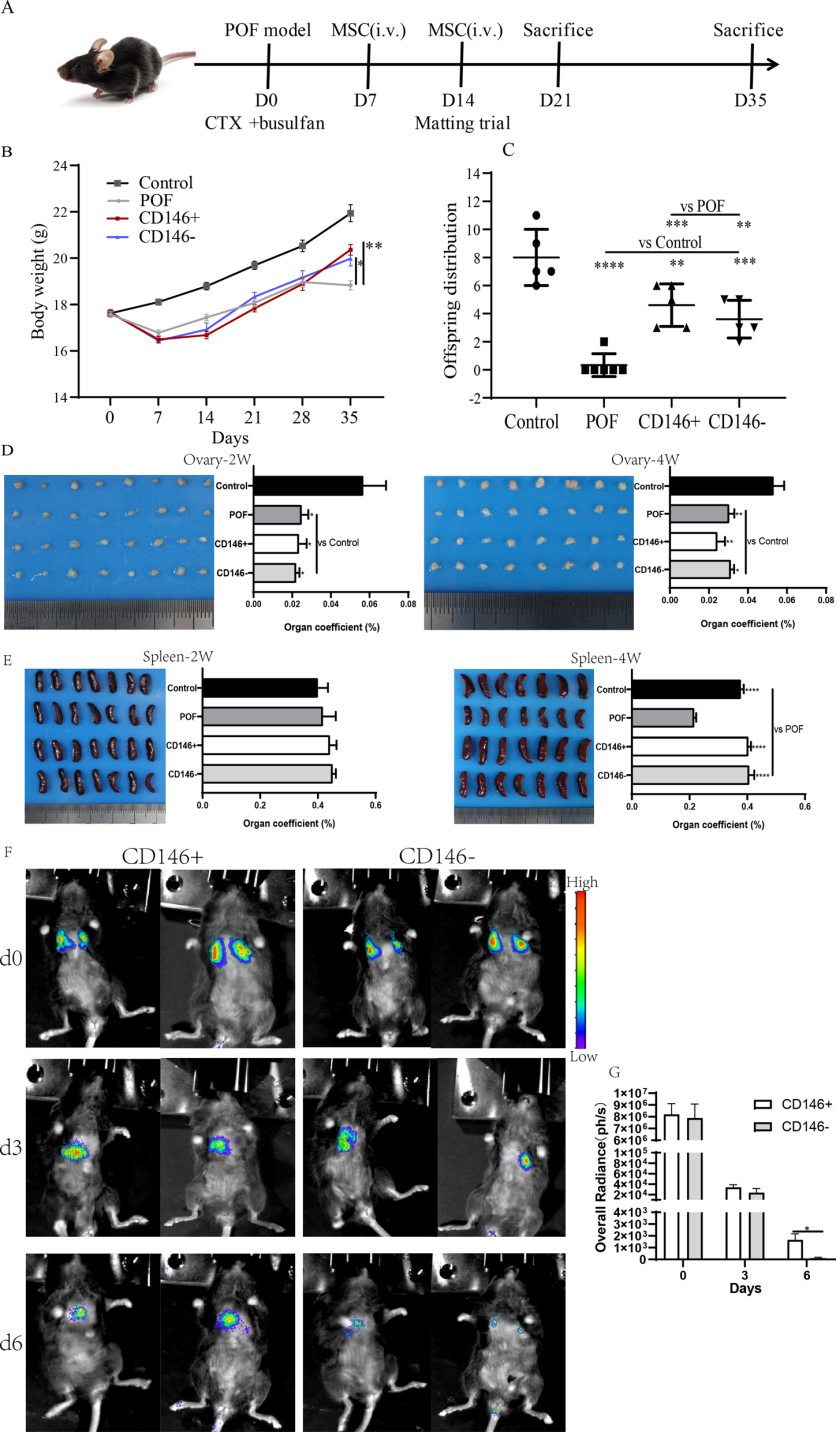
The body weight, litter size, organ coefficient and distribution in POF mice after CD146 +/− MSCs treatment. **A** The time scale of animal experiment was marked. The stem cells were injected on days 7 and 14 after modeling, and the mouse mating experiment was conducted on day 14, the mice were killed for the first time in 21 days and for the second time in 35 days. **B** The body weight changes of mice. **C** The litter size of mice. **D**, **E** Ovaries and spleens coefficient at two weeks (2 W)and four weeks (4 W)after the first stem cell injection. **F**, **G** In vivo imaging of CD146 +/− MSCs at days 0, 3, and 6 after intravenous tail vein injection. As can be observed in the images and from the radiancestatistics, CD146 + MSCs lived longer and more in the lungs than CD146 - MSCs

Mouse body weight was monitored weekly. The average body weight of each group was about 18 g at experiment beginning. Treatment of mice with chemotherapy drug resulted in the decrease in body weight. Then the body weight of the mice gradually recovered. The average body weight was significantly higher in the MSCs-treated group than in the POF group after MSCs transplantation for two weeks. Both CD146 + MSCs and CD146 - MSCs exhibit effect in maintaining body weight. However, there was no significantly difference between CD146 + MSCs and CD146 - MSCs. The therapeutic effect of both cell types on POF was evaluated through the mating test of fertility. The average fertility of the control group was 8.00 ± 1.79, the POF group was 0.33 ± 0.80, the CD146 + MSCs group was 4.60 ± 1.36, and the CD146 - MSCs group was 3.60 ± 1.20. The ovarian and spleen tissue were weighed to calculate the organ coefficients. Compared with the POF group, there was no significant difference in improving ovarian weight between the CD146-positive and CD146-negative groups (Fig. 3D). At the end of treatment, the spleens of the POF model group were seriously atrophied (Fig. 3E). The litter size is 8 ± 0.9 in the control group, 4.6 ± 0.7 in the CD146 + MSCs group, 3.6 ± 0.6 in the CD146 - group, while the POF group almost had no offspring (0.3 ± 0.3). Even though MSCs therapy significantly increased litter size, its restoration could not achieve the level of the control group. MSCs also improved spleen atrophy caused by chemotherapy drugs. However, there was no significant difference between the CD146 +/− MSCs groups in this regard. The distribution of MSCs was further explored by labeling with adenovirus carrying luciferase gene in mice (Fig. 3F), and most of the CD146 +/− MSCs are stayed in the lung within six days. The MSC radiance on day d3 was about 20-fold lower than that on day0 (Fig. 3G), suggesting that most of the stem cells were metabolized (Additional file 2: Figure S2). The radiance of the CD146 - MSCs group was slightly lower than that of the CD146 + MSCs group on day3, but was significantly lower than that of the CD146 + MSCs group on day 6. It is suggested that CD146 + MSCs could survive and maintain longer time in the lung.

### MSCs transplantation increases the number of ovarian follicles, promotes ovarian cell proliferation, and restores serum estrogen levels

**Fig. 4:**
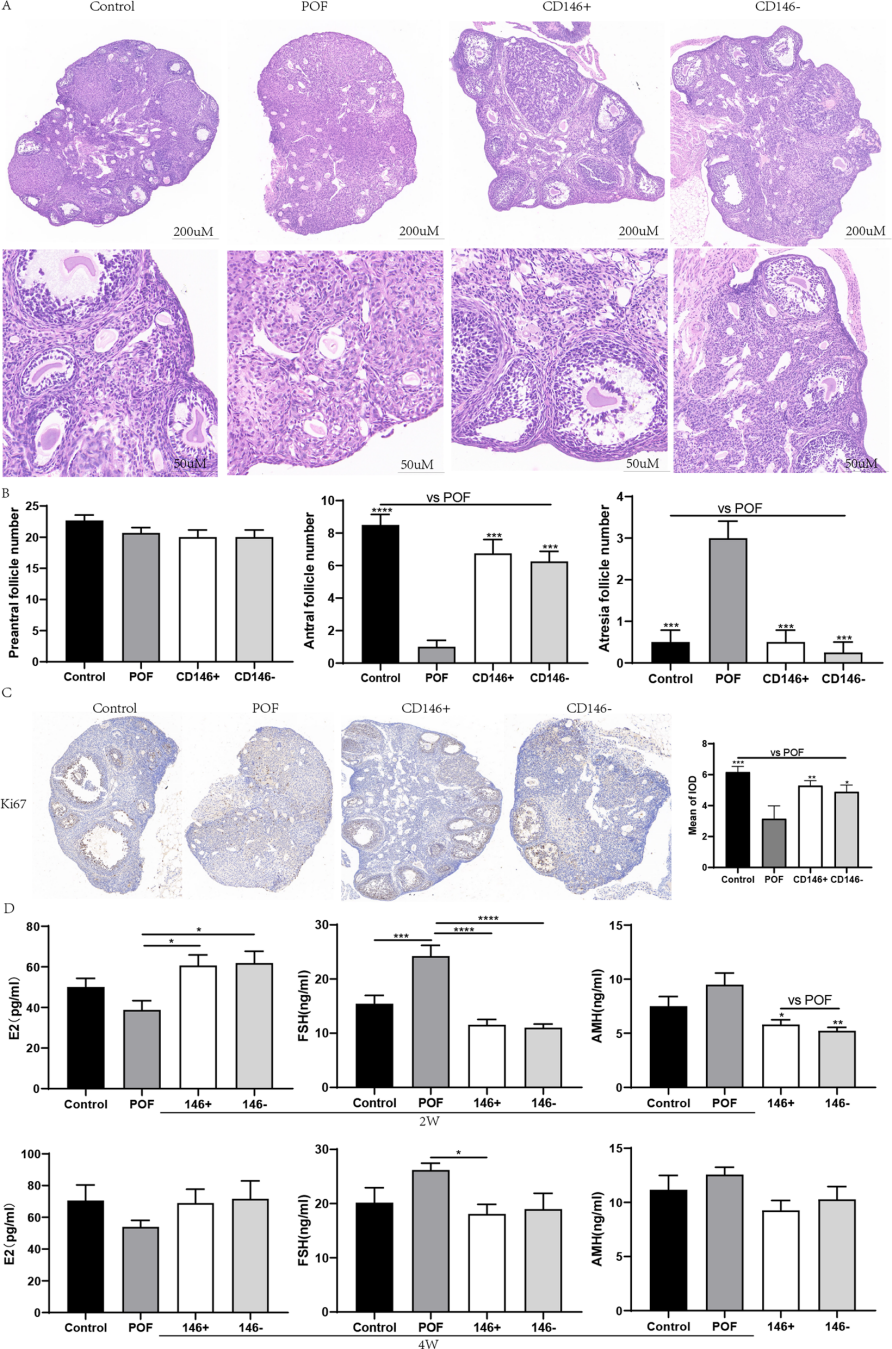
Impacts of CD146 +/− MSCs on ovarian function and estrogen secretion levels in POF mice. **A** HE staining of mouse ovary. **B** Different types of ovarian follicles were observed from the ovaries of each group. **C** Ki67 immunohistochemistry and IDO value by IPP software. **D** Estrogen (E2, FSH, AMH) levels of each group in peripheral blood at 2 W and 4 W

HE staining results showed that MSC therapy significantly improved the number of sinus follicles (Fig. 4A). However, there was no significant difference between the groups with regard to the structure of sinus follicles (primordial follicles or primary follicles) (Fig. 4B). The MSCs therapy group had a significantly higher number of sinus follicles (*P* < 0.01). The proliferation of ovarian cells was further validated by Ki67 staining. It was observed few proliferation cells in the POF group, whereas in the control and MSCs treatment group, more positive ovarian cells were stained (Fig. 4C). Compared with POF group, the staining areas in the control group (*P* < 0.001), the CD146 + MSCs group (*P* < 0.01), and the CD146 - MSCs group (*P* < 0.05) were concentrated on the granulosa cells of large antral follicles, indicating that the follicles in these groups developed well. The POF group displayed substantial loss of primordial follicles, but not of mature follicles.

We detected the serum estrogen levels at 2 W and 4 W post-transplantation of MSCs (Fig. 4D). At 2 weeks post-transplantation, the E2 concentration in the MSCs-treated group was significantly higher than that of the POF group (*P* < 0.05), whereas the FSH concentration in the MSCs-treated group was significantly lower than that in the POF group (*P* < 0.0001). The POF group also had a higher level of AMH, which was associated with a higher number of pre-sinus follicles and excessive mobilization of the follicle pool. At 4 weeks post-transplantation, MSCs treatment enhanced the recovery of hormones to a certain degree, but there was no significant difference between the groups. The exception was that estrogen levels in the CD146 + MSCs group were significantly lower than those in the POF group (*P* < 0.05). These results showed that UC-MSCs transplantation could significantly inhibit granulosa cell apoptosis, improve estrogen secretion and ovarian histological morphology, reduce follicular atresia, and increase the number of antral follicles after chemotherapy.

### CD146 + MSCs treatment improved recovery of chemotherapy-induced immunologic dysfunction

**Fig. 5:**
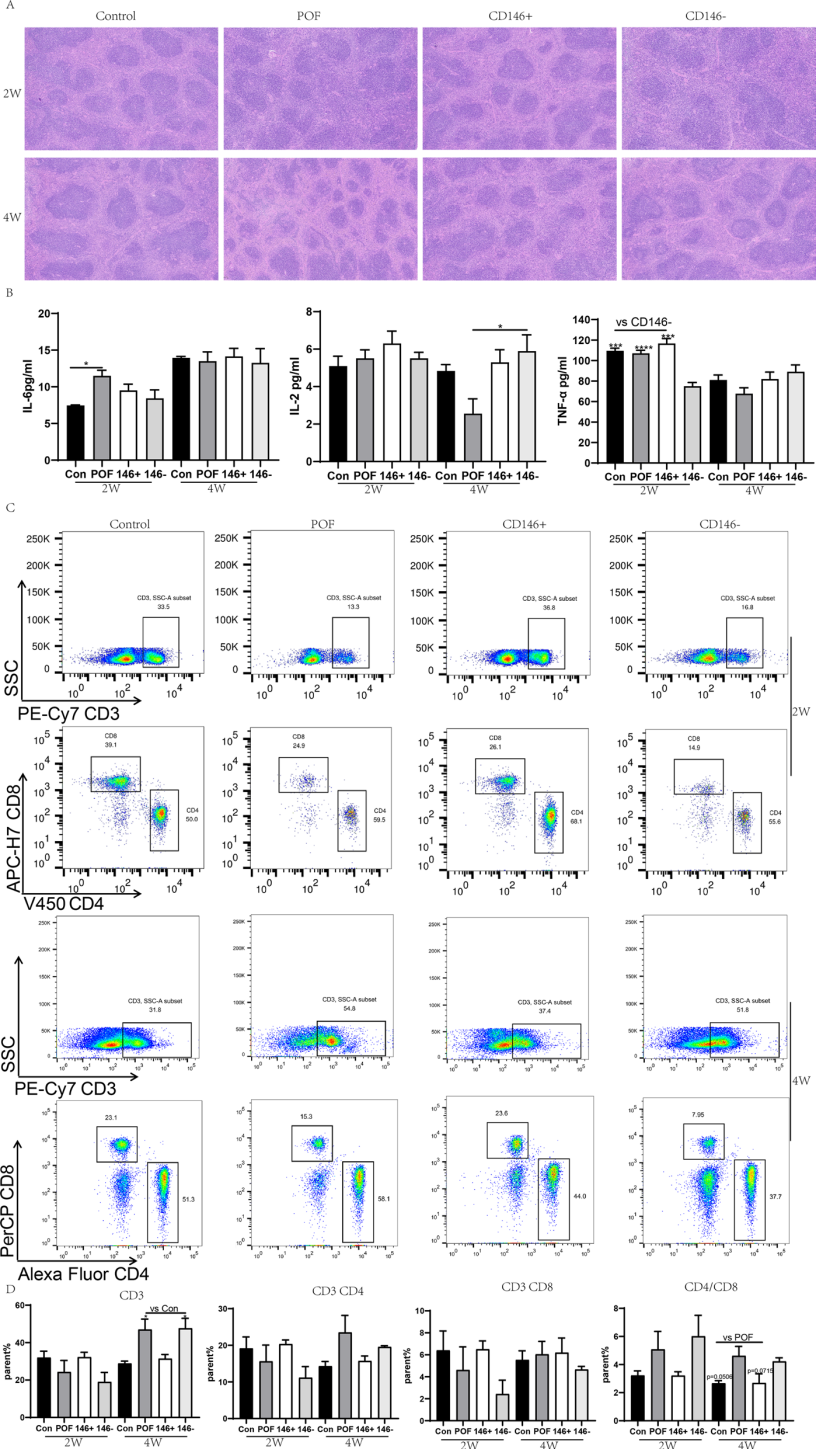
Impacts of CD146 +/− MSCs on spleen morphology and lymphocyte percentage in POF Mice. **A** HE staining of mouse spleen. **B** The levels of cytokine (IL-6, I-L2, TNF-α) in peripheral blood at 2w and 4 W. **C** The ratio of CD3, CD4 and CD8 in peripheral blood of mice at 2 W and 4 W

MSCs treatment (with either CD146 + MSCs or CD146 - MSCs) significantly protected mice from spleen shrinkage induced by chemotherapy agents. The HE staining results indicated that structural spleen abnormalities were significantly fewer in the POF group at 2 weeks post-transplantation, and the shrunken spleens had recovered at 4 weeks post-transplantation (Fig. 5A).

The inflammatory cytokine IL-6 in serum was assayed with ELISA, revealing that serum IL-6 levels were significantly higher in the POF group than in the control group at 2 weeks post-transplantation (*P* < 0.05) (Fig. 5B). This suggests that inflammation from chemotherapy affected POF mice. Therefore, it appears that MSCs treatment could reduce the release of inflammation cytokines in POF models. In addition, the level of TNF-α in the CD146 + MSCs group was significantly lower than in the other three groups (*P* < 0.001).

The percentage of T lymphocytes in peripheral blood was analyzed with flow cytometry (Fig. 5C). At 2 weeks post-transplantation, the percentage of CD3, CD4, and CD8 positive cells in POF mice was lower than that in the control group. Treatment of POF mice with CD146 + MSCs prevented the chemotherapy-induced decrease in the percentage of lymphocytes, whereas CD146 - MSCs treatment did not show a significant protection effect (Fig. 5D). The ratio of CD4/8 lymphocytes in the CD146 + MSCs group was significantly higher than that of the control group or CD146 - MSCs group. In the POF group, at 4 W, percentages of CD4 and CD8 had returned to the baseline. However, the percentage of CD3 + lymphocytes in POF mice compared with the control group and CD146 + MSCs group was significantly higher (*P* < 0.05). The CD4/8 ratio in the POF group remained lower than in the control group (*P* = 0.0506) or the CD146 + MSCs group (*P* = 0.0715). The above results suggest that the treatment effects of CD146 +/− MSCs in improving chemotherapy-induced immunologic dysfunction are different and that the CD146 + MSCs are more efficacious in regulating immunologic dysfunction.

### CD146 +/− MSCs improved ovarian function through PPAR and cholesterol metabolism pathways

**Fig. 6:**
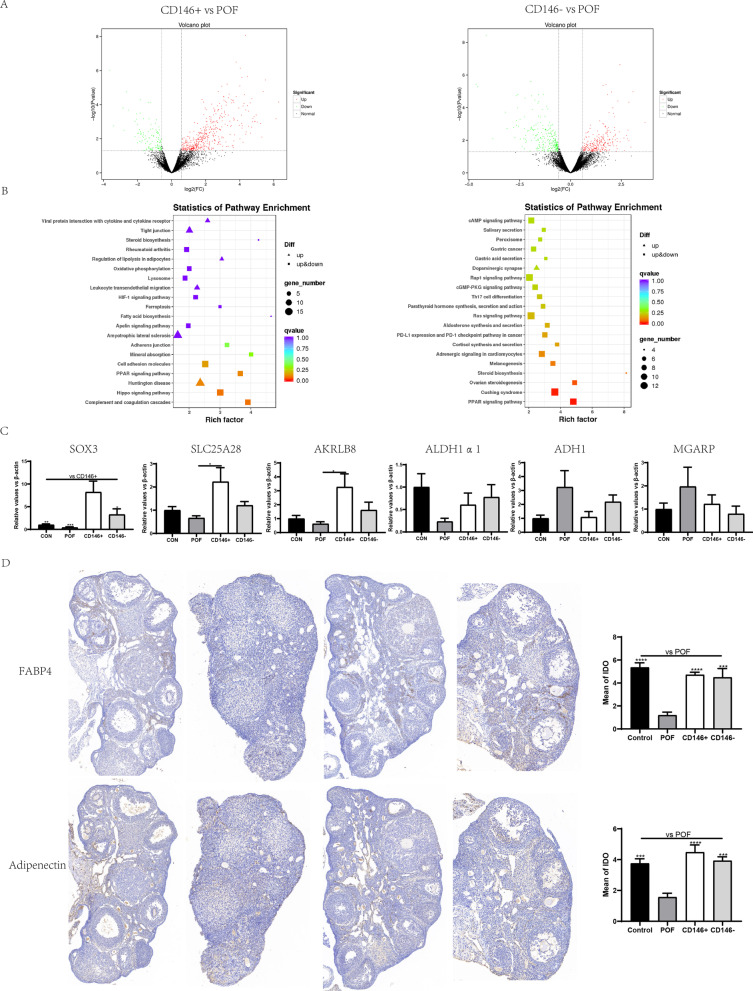
Transcriptome sequencing of ovarian tissues and verification. **A** mRNA-sequencing was used to detect the differential genes between groups. **B** KEGG pathway enriched by differential genes. **C** qPCR was used to verify the sequencing results. **D** The immunohistochemistry of FABP4 and adiponectin, and IPP software was used to calculate the IDO values of each group

The mechanisms that allow CD146 +/− MSCs to repair chemotherapy-drug-induced POF were further elucidated. The specimens from two weeks after MSC transplantation were analyzed by RNA sequencing. There was a total of 573 different genes, including 449 up genes and 124 down genes, between the CD146 + MSCs group and POF group. And 511 differential genes were detected between the CD146 - MSCs and POF groups, including 264 up-regulated genes and 247 down-regulated genes. We used KEGG analysis to look at the gene differences between the CD146 +/− MSCs group and the POF group in order to understand differences in their potential function and possible pathways. The KEGG analysis showed that DEGs (146 + MSCs vs the POF group) were significantly enriched in the Complement and Coagulation cascades, Hippo signaling pathway, Huntington Disease, and PPAR signaling pathway. DEGs (CD146 - MSCs vs POF group) are primarily associated with the PPAR signaling pathway, Cushing’sSyndrome, ovarian steroid genesis, and steroid biosynthesis (Fig. 6B).

Several significant genes were selected and further validated with the qPCR method, including Sox3, Lpl, Slc25a28, Adipoq, Fabp4, AKRLB8, Mgarp, and Adh1. The differential expressed genes in CD146 + MSCs and CD146 - MSCs were related to repair of ovarian function through increasing genes such as Sox3, Lpl, Slc25a28, and AKRLB8 and decreasing genes such as Mgarp and Adh1. Compared with the POF group, the Sox3 gene was about 20 times more prevalent in the CD146 + MSCs group and about 8 times more prevalent in the CD146 - MSCs group (Fig. 6C). Furthermore, Fabp4 and Adipoq gene expression was validated by immunohistochemical staining (Fig. 6D). The result indicated that Fabp4 was mostly expressed in the interstitial part of ovarian tissue, and Adipoq was mostly expressed in the center of follicles.

## Discussion

The POF mouse model was established using cyclophosphamide, which is commonly used for cancer therapy as a clinical chemotherapy drug. Chemotherapy drugs cause cell damage of dividing cells and have a series of side effects on the immune and reproductive systems [36]. Using a cyclophosphamide-induced POF model, multiple studies have shown that mesenchymal stem cells derived from various sources, such as bone marrow, amniotic tissue, adipose tissue, menstrual blood, and umbilical cord tissue, can improve ovarian damage and endocrine function and repair ovarian tissue structure [37–39]. In this study, by using a mouse POF model which is induced by cyclophosphamide and busulfan, we found that both CD146 + MSCs and CD146 - MSCs subpopulations have therapeutic effects on POF. However, CD146 + MSCs have a slight advantage over CD146 - MSCs. After MSCs therapy, both subpopulations are able to restore the function of damaged ovarian tissue and follicle development and allow mice to produce offspring. It seems that CD146 + MSCs and CD146 - MSCs have similar therapeutic efficiency in ovarian follicle development, but CD146 + MSCs exhibit greater proliferation ability and stronger immune-modulating activity.

In vitro experimental results show that CD146 + subsets had stronger proliferation characteristics compared with CD146 - MSCs. The sequencing results show that both CD146-positive and CD146-negative MSCs have the regenerative characteristics with growth factor secretion related to tissue repair, which may be the reason why there was little difference between the two subsets in the various groups with regard to repairing ovarian damage. These results also support the previous reports by Bowles et al. that different gene expression between CD146 +/− subsets mainly centers on immune regulation, but that there is no difference in secretion of growth regulators [15]. Both cyclophosphamide and busulfan have higher ovarian toxicity and cause follicle consumption destruction, vascular injury, and fibrosis of the ovarian cortex. A previous study indicated that 42% of women patients receiving treatment with an alkylating agent suffered from POF [40]. Cyclophosphamide could directly induce apoptosis of oocytes leading to POF through activation of primordial follicles and acceleration of primordial follicle consumption [41]. In this study, HE staining showed that follicular development stopped at the stage of primary follicles, leading to fewer sinus follicles in POF ovaries. Consistent with damage to sinus follicles, serum AMH levels were also increased in the POF group. Because AMH is produced by pre-sinus follicles, this provides evidence that the POF group had more pre-sinus follicles in damaged ovaries [42]. Dysregulation of E2 and FSH feedback is characteristic in mouse POF models. Abnormally elevated FSH suggests that the ovary's response to FSH is insensitive and ovarian reserve is decreased [43]. These comprehensive data confirmed that busulfan and cyclophosphamide induce POF through excessive activation of primordial follicles, prompting abnormity follicular atresia and eventually leading to premature ovarian failure. Follicular development is regulated by steroid hormones and many other growth factors. Ovarian steroidogenesis requires two main steroidogenesis theca cells and granular cells for the glandular structure of collaborative interaction [44]. First, the LH receptor of the theca cells receives the LH signal to convert cholesterol into progesterone, and then the FSH receptor of the granulosa cells binds to FSH, thereby increasing the specific enzyme to convert androgen into estrogen [45].

It seems that recovery from chemotherapy-induced POF is dependent on anti-apoptosis and angiogenesis. Mesenchymal stem cells improve the follicular microenvironment to recover ovarian function in mice with premature ovarian failure [46]. Emerging evidence indicated multiple mechanisms including exosome release, antioxidative substance and growth factor secretion contribute to the therapeutic effect of mesenchymal stem cells on POF. The mesenchymal stem cells release exosomal miR-644-5p, miR-144-5p to inhibit ovarian granulosa cell apoptosis through targeting p53 or Pten [47, 48], In the chemotherapy-induced rat POF model, the NGF/TRK pathway is involved in and contribute to the amelioration effect of mesenchymal stem cells on POF [49]. In this study, Ki67 immunostaining results confirmed that the control group and MSCs treatment group had superior proliferation ability of local follicle granulosa cells, while more apoptotic cells were observed in the POF group. In vivo tracing experiments on MSCs showed that the CD146 +/− subsets were mainly distributed in the lungs of mice after magnetic bead sorting. But animal imaging experiments showed that the CD146 + MSC group persisted in the mouse body for a longer time. Currently, it is hard to analyze the UC-MSC-derived exosomal miRNA and growth factors in the mouse POF model. But the different therapeutic effect of CD146 +/− MSCs subpopulation might be associated their survival period in vivo and the active substances they released.

Transcript profile might clarify the mechanisms that CD146 +/− MSCs be beneficial to the POF. The data revealed that MSCs repair ovarian function mainly through the PPAR pathway, which plays an important role in improving ovarian steroid metabolism and lipid metabolism. Previous research has found that cyclophosphamide induces premature ovarian failure by blocking the cholesterol biosynthesis pathway in which peroxisome proliferator-activated receptor (PPAR) is involved and mediates its blocking [50]. The PPAR family of transcription factors has proved to link lipid/glucose availability and long-term metabolic adaptation in POF [51]. Transcriptome data also demonstrate that CD146 + MSCs treatment significantly activates PPAR signaling in POF models. Our findings suggest that MSCs treatment restores ovarian function through increasing AMH levels and diminishing ovarian inflammation. This takes place predominantly via upregulation of PPAR-γ and leads to inhibition of provoked inflammatory cytokines. Our data confirmed that MSCs therapy regulates cholesterol and steroid biosynthesis pathways by modulating their key enzymes and prompting them to restore ovarian endocrine function.

Stem cell therapy can significantly improve the spleen atrophy caused by chemotherapy. In mouse POF models, chemotherapy induces spleen hyperfunction early and then causes the spleen to gradually shrink. Both CD146 + MSCs and CD146 - MSCs protect the spleen from chemotherapy-induced atrophy. Furthermore, it is interesting to note that the percentage of lymphocytes in peripheral blood were significantly recovered in the CD146 + MSCs group, while CD146 - MSCs had no effect on lymphocyte recovery at 2 W. The CD3 expression in the POF group was decreased at 2 weeks post-transplantation and significantly increased at 4 weeks post-transplantation. The change trends in the CD146 - MSCs group were similar to those in the POF group. This indicates that two subsets of CD146 +/− MSCs regulate chemotherapy-induced immune dysfunction differently. The mice in the CD146 + MSCs group recovered their immune function better and faster. The in vitro sequencing of CD146 +/− MSCs also showed that the CD146 + subset secreted low levels of pro-inflammatory factors. These results indicate that the CD146 + subset is a group of cells with superior immune regulatory characteristics. At 4 weeks post-transplantation, the ratios of CD4 and CD8 in the POF group were higher than those in the control group or CD146 + MSCs group, which might be because the immunosuppressive effect of cyclophosphamide disappeared and there was a compensatory increase in immune cells from new tissues in immune organs. Furthermore, HE staining showed that cyclophosphamide exposure results in spleen structure disruption, a higher spleen index, and changes to the lymphocyte percentage in peripheral blood. This result is consistent with previous studies in which the immunosuppressive effect of cyclophosphamide gradually disappeared over time, leading to a compensatory increase in the spleen index [52]. A high dose of cyclophosphamide can affect lymphocyte expansion, reducing their number and percentage. The effect of cyclophosphamide on the spleen of mice was significantly correlated with the action time and the administered dose.

## Conclusions

The CD146 +/− subsets do not show much difference in the repair of chemotherapy-induced ovarian damage. CD146 + MSCs have a weak advantage over CD146 - MSCs, which may be related to their better proliferation characteristics and longer retention time in vivo. The CD146 +/− subsets display differences in the regulation of immune function. The CD146 + MSCs can strongly inhibit T cell proliferation and secretes lower levels of inflammatory factors in vitro. The UC-CD146 + MSCs might have the advantages in regulating function of immune cells and for treatment of immunodiseases.

## Supplementary Information

**Additional file 1.** The percentages of CD146 + MSCs before and after separation.** A** The proportion of CD146 + MSC before isolation.** B** The CD146+ ratio of CD146 + subgroups after isolation and continuous culture for 24 h and 48 h.** C** The foldchange of CD146+ ratio in cultured MSCs without selection for several generations (n = 3).

**Additional file 2.** The bioluminescence imaging of organs on day 8 after MSCs injection.

## Data Availability

The raw mRNA-seq data reported in this paper have been deposited in the Genome Sequence Archive in National Genomics Data Center (GSA: CRA006709) that are publicly accessible at https://ngdc.cncb.ac.cn/gsa. The other data used or analyzed during the current study are available from the corresponding author on reasonable request.

## Abbreviations

MSCs: Mesenchymal stem cells
POF: Premature ovarian failure
UC: Umbilical cord
RT-PCR: Real-time polymerase chain reaction
PBS: Phosphate-buffered saline
FBS: Fetal bovine serum
H&E: Hematoxylin–eosin staining
FSH: Follicle-stimulating hormone
E2: Oestradiol
AMH: Anti-Mullerian hormone
IL-6: Interleukin-6
IL-2: Interleukin-2
TNF-α: Tumor necrosis factor Alpha
Elisa: Enzyme-linked immunosorbent assay
CXCL: CXC chemokine ligands
KEGG: Kyoto Encyclopedia of Genes and Genomes
PPAR: Peroxisome proliferators-activated receptors
