# Supplementary figures and images for: Comparison of CD146 +/− mesenchymal stem cells in improving premature ovarian failure
Source: Stem Cell Res Ther. 2022 Jun 21;13:267. doi: 10.1186/s13287-022-02916-x (PMC9209844; doi:10.1186/s13287-022-02916-x)

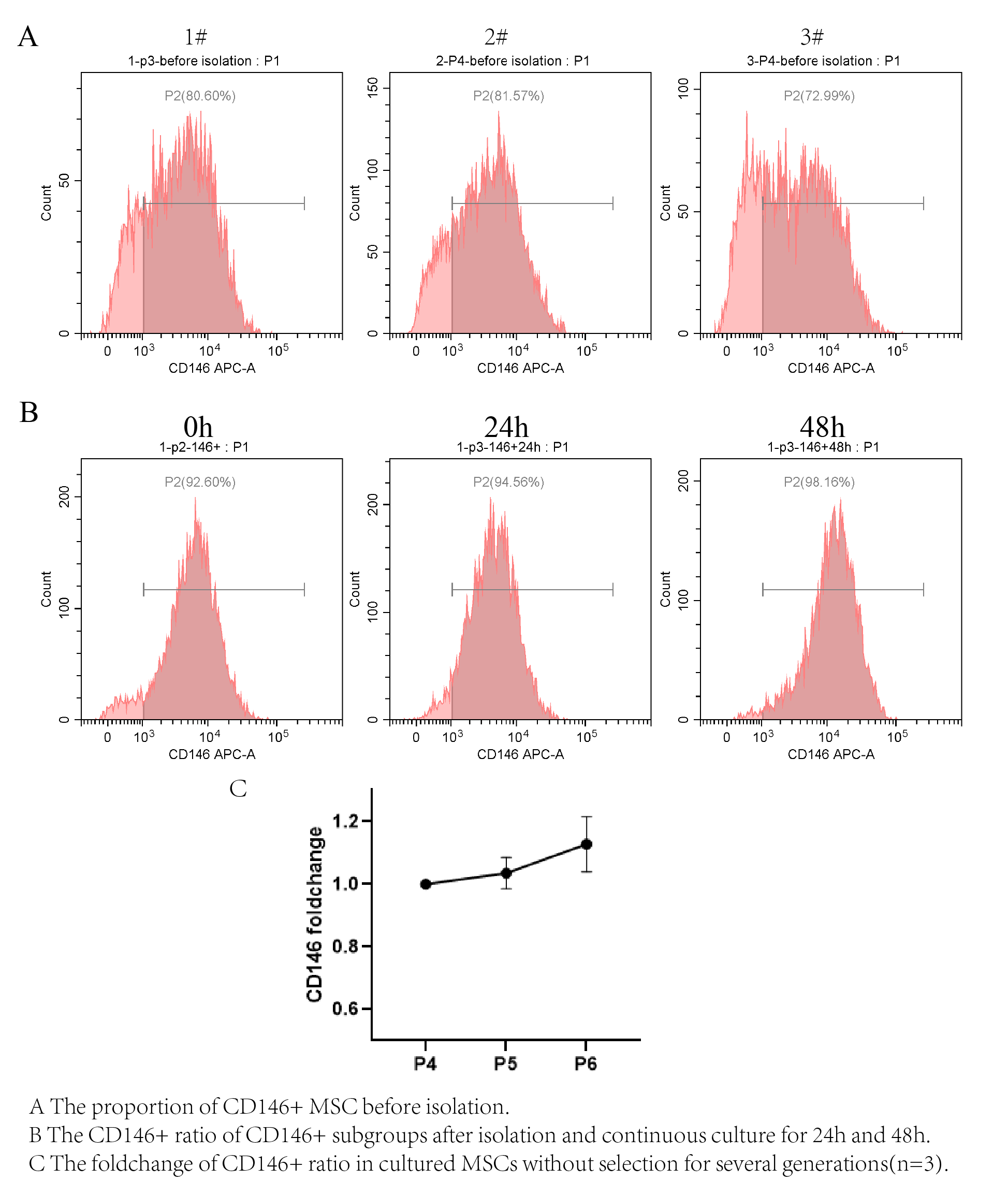

Supplement: Supplementary file 1 — Additional file 1. The percentages of CD146 + MSCs before and after separation. A The proportion of CD146 + MSC before isolation. B The CD146+ ratio of CD146 + subgroups after isolation and continuous culture for 24 h and 48 h. C The foldchange of CD146+ ratio in cultured MSCs without selection for several generations (n = 3). [file 13287_2022_2916_MOESM1_ESM.png]

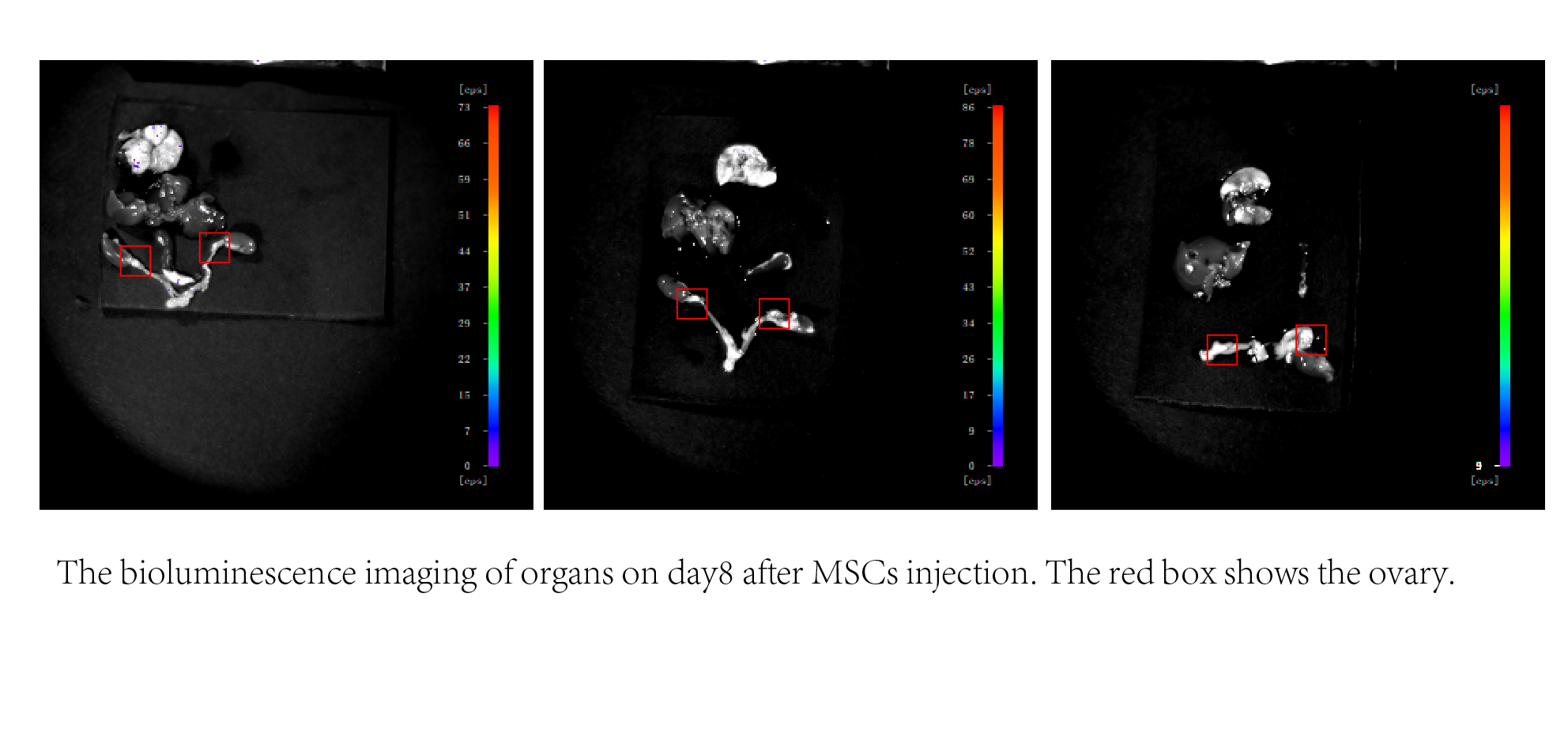

Supplement: Supplementary file 2 — Additional file 2. The bioluminescence imaging of organs on day 8 after MSCs injection. [file 13287_2022_2916_MOESM2_ESM.png]
